# Left inferior-parietal lobe activity in perspective tasks: identity statements

**DOI:** 10.3389/fnhum.2015.00360

**Published:** 2015-06-30

**Authors:** Aditi Arora, Benjamin Weiss, Matthias Schurz, Markus Aichhorn, Rebecca C. Wieshofer, Josef Perner

**Affiliations:** ^1^Department of Psychology, University of SalzburgSalzburg, Austria; ^2^Center for Neurocognitive Research, University of SalzburgSalzburg, Austria

**Keywords:** identity, false belief, episodic memory, visual perspective taking, fMRI, IPL, overarching function

## Abstract

We investigate the theory that the left inferior parietal lobe (IPL) is closely associated with tracking potential differences of perspective. Developmental studies find that perspective tasks are mastered at around 4 years of age. Our first study, meta-analyses of brain imaging studies shows that perspective tasks specifically activate a region in the left IPL and precuneus. These tasks include processing of false belief, visual perspective, and episodic memory. We test the location specificity theory in our second study with an unusual and novel kind of perspective task: identity statements. According to Frege's classical logical analysis, identity statements require appreciation of modes of presentation (perspectives). We show that identity statements, e.g., “the tour guide is also the driver” activate the left IPL in contrast to a control statements, “the tour guide has an apprentice.” This activation overlaps with the activations found in the meta-analysis. This finding is confirmed in a third study with different types of statements and different comparisons. All studies support the theory that the left IPL has as one of its overarching functions the tracking of perspective differences. We discuss how this function relates to the bottom-up attention function proposed for the bilateral IPL.

## Introduction

There is growing evidence that the dorsal part of the left temporo-parietal junction (TPJ), which overlaps with the left inferior parietal lobe (IPL), is reliably activated by perspective tasks (Goel et al., 1995; Ruby and Decety, 2003). Perspective tasks are tasks that require tracking of (potential or actual) perspective differences^1^. Findings from cognitive development indicate that these tasks share a common cognitive basis. They are mastered around the age of 4 years. Brain imaging studies of perspective tasks also point to a common neural basis. Existing evidence suggests regional specificity (Kanwisher, 2010) of different kinds of perspective tasks activating the left IPL^2^. Our aim is to test this specificity hypothesis in three steps. In the first step we carry out a meta-analysis of existing data from three different kinds of perspective tasks to test the regional specificity hypothesis. Partial activation overlap of the different kinds of tasks within left IPL counts in favor of the hypothesis. In the second step we test the hypothesis further with the prediction that a novel and unusual perspective task, processing identity statements, should activate within the region identified by the meta-analysis. In a third step we confirm this finding with novel stimulus material. To carry through with this project we need to be more specific about what perspective tasks are and about the criteria that define the region of overlap, for which we adopt the overarching view proposed by Cabeza et al. (2012).

## What are perspective tasks?

In response to this question we follow the intuition elaborated by Perner et al. (2003), who links the notion of perspective to the notion of representation and modes of presentation. A representation represents something (object, target) as being in a certain way (content). The content provides a perspective of the target. Hence, if two representations represent the same target (e.g., the spatial relation between objects A and B) but differ in their content, i.e., how they represent the target as being (“A is in front of B” vs. “A is behind B”) then we face a perspective difference. Similarly, if one person individualizes an entity as a *mouse*, another person the same entity as an *animal*, they differ in how they think of the same target object. Psycholinguists express this point by saying that the choice of label for an object puts a different perspective on that object (see Clark, 1997; Tomasello, 1999). In general, a perspective task can be characterized as a task where one becomes aware of the distinction between the target and content. We now need to show that this can cover the different cases in which all visual perspective tasks are thought to play a role.

### Visual perspective

If two people look at different scenes their visual representations are likely to differ because they see different scenes and not because they have different visual perspectives of the same scene. In contrast, if they stand side by side looking at the same scene they see the same things in the world but their visual representations still differ. Since they are looking at the same scene that difference cannot be attributed to a difference in the scenes they are looking at (the target) but only to how that single scene presents itself differently to them due to their different viewing positions. In the developmental literature children's understanding of perspective in this sense has been captured by the notion of Level 2 perspective taking (Masangkay et al., 1974; Flavell et al., 1981). At around 4 years of age children become able to understand that people who look at the same objects may see them related in different ways due to their different viewing position. The classic example is a simple drawing of a turtle positioned on a table between experimenter and child, who face each other across the table. Children before the age of 4 years understand that the turtle “stands on its feet” when its feet are pointing toward the child, and that it is “lying on its back” when the drawing has been turned by 180°. However, when asked whether the experimenter sees the turtle as standing on its feet or lying on its back they cannot give a correct answer until around 4 years of age. In contrast, much younger children have no problems with Level 1 perspective taking tasks, which test the understanding that people may see different things from different vantage points. For instance, if on a piece of paper, e.g., a car is drawn on one side and a lion on the other side, children correctly point out that the experimenter can see the car when they can see the lion.

Unfortunately, brain imaging studies do not systematically observe this distinction between Levels 1 and 2 tasks. Most of them contrast questions about what another person can see with what the participants themselves can see. Although this often only requires a Level 1 understanding, it is still likely that instruction to pay attention to what others see naturally triggers Level 2 perspective taking processes.

### False belief

The false belief test (Wimmer and Perner, 1983) has become the most popular way of assessing understanding and processing of other people's mental states both in developmental (Wellman et al., 2001) and brain imaging research (Saxe and Kanwisher, 2003). Brain imaging studies present short vignettes in which people develop a false belief (e.g., Aichhorn et al., 2009): “Julia sees the ice cream van go to the lake. She doesn't see that the van turns off to the town hall. Therefore, Julia will look for the ice cream van at the… lake/town hall?” To understand that Julia is mistaken about the location of the ice cream van one has to understand that she represents the van as being at the lake, while we know that it is at the town hall. Both, Julia and we represent the current location of the van (target) but she represents it as being at the lake while we represent it as being at the town hall. This is a difference in content hence a difference in perspective.

In contrast most imaging studies use the so-called “false photo” task^3^ (originally designed for children; Zaitchik, 1990), e.g., “Julia takes a picture of the ice cream van in front of the pond. The ice cream van moves to the market place; the picture gets developed. In the picture the ice cream van is by the… pond/market place?” (Aichhorn et al., 2009). Although this task parallels in many ways the belief task—an object changes location and a representation of the object in its original location (photo/belief) persists—there are crucial differences. Unlike the belief the photo is not false and, unlike the belief, one does not have to understand the photo as giving a differing perspective on the object's location from its actual location. One just has to describe where the object is in the photo (notice: one could not ask “In Julia's belief the ice cream van is … ?”).

### Episodic memory

Episodic memory is defined in Tulving's tradition by Wheeler et al. (1997) as re-experiences of earlier experiences. Re-experience requires tracking of perspective. When simply experiencing an event one just takes in the event without reflecting on the fact that one has had an experience. In contrast, when re-experiencing a past event one has to understand that the experience one currently has provides but a view (perspective) of an actual past event. Without this awareness one would either mistake the re-experience for an actual experience resulting in severe delusion, or one would mistake it for an experience of an imagined, fictional event. In neither case would it count as remembering the past.

The strictest way to test for episodic memory is the remember-know judgment (Tulving, 1989). When able to retrieve a learned item or able to recognize it, participants are asked to judge whether they really remember the item, i.e., can relive their experience, or whether they just know that the item had been presented. Unlike knowing of an event the critical element of remembering an event is the double awareness of re-experiencing the event and of the fact that the event happened in one's past. In order not to mistake the re-experience as experiencing the same event again (Martin, 2001) one has to understand the ongoing re-experience as providing a perspective on something that has happened in the past.

### False signs

This task has been developed for children (Parkin, 1994) and was adopted for brain imaging by Aichhorn et al. (2009), e.g., The ice cream vendor's sign points to the lake. The ice cream van goes to the town hall without changing the sign. According to the sign post the ice cream van is at the… lake/town hall?” The false sign vignettes share with the false belief vignettes misinformation or misconception about the current state of things. In the belief vignette Julia thinks the van is at the lake, and in the false sign vignette the sign shows that the van is at the lake, when it really is at the town hall. Both vignettes differ from the “false photo” vignettes in this respect. The photo does not show where the ice cream van is, and participants are asked where in Julia's photo the van is. As pointed out earlier, this question is not possible for Julia's belief (Where in Julia's mind is the ice cream van?) and it is not possible for the false sign (Where in the sign is the ice cream van?). The two imaging studies that used false sign vignettes tested whether these vignettes activated the same brain regions as false beliefs in contrast to the “false photo” vignettes.

## Commonality of perspective tasks

### Developmental synchrony

The four kinds of perspective tasks listed above are those for which we could find brain imaging data. All of them have been used in child appropriate versions in developmental studies. They all tend to be mastered between the age of 3–5 years (e.g., episodic remembering: Perner and Ruffman, 1995; Naito, 2003). Moreover, several studies have used the false belief task together with other perspective tasks and consistently found correlations between these tasks when controlling for differences in age and verbal intelligence (for overview see Perner and Roessler, 2012). In particular, passing the false belief task correlates with passing the level 2 visual perspective task (Hamilton et al., 2009—also in children with autism) and with passing the false sign task (Parkin, 1994; Bowler et al., 2008—also in children with autism; Sabbagh et al., 2006; Leekam et al., 2008; Iao and Leekam, 2014). Another perspective task used with children, which has not been used for brain imaging, is the appearance reality task (Flavell et al., 1983), in which children are explicitly asked what a deceptive object (a piece of sponge that looks like a rock) looks like and what it really is. Children's ability to draw this distinction also correlates with passing the false belief task (Gopnik and Astington, 1988; Taylor and Carlson, 1997; Courtin and Melot, 2005).

### Cerebral overlap: the overarching view

Many of the developmental perspective tasks have been used in brain imaging experiments on adults. We now look for evidence whether their common development is also reflected in shared brain activity. A strict criterion for sharing brain activation would be activation overlap of all perspective tasks. This may, however, be an overly conservative criterion as Cabeza et al. (2012) argued for a similar case. Instead of looking for complete overlap they proposed the “overarching function” view that allows for subdivisions within a broader brain region. The broad region (in our case, the left IPL) has a global, overarching function (tracking perspective) and its various sub-regions mediate different aspects (false beliefs, visual perspectives, etc.) of the global function. The expected pattern of finding is that each perspective task should activate the broad region and partially overlap with activations by other perspective tasks. To check whether existing data support this view we extended an existing meta-analysis for false belief studies and visual perspective taking by Schurz et al. (2013) by also including episodic memory studies testing for remember-know judgments.

## Study 1: meta-analysis

For false belief studies and visual perspective studies we used the meta-analysis data from the work by Schurz et al. (2013) based on 25 false belief and 14 visual perspective taking (vPT)^4^ studies. To this we added a meta-analysis of episodic memory (EM) studies that contrast items judged as “remembered” or “recollected” (the sense of being able to re-experience the learning phase) with items judged as just “known” or “of high confidence familiarity” (the sense of the item being old without a re-experience of learning the item). We found 16 studies that make the relevant contrasts (see details in Table S1 in supplementary material).

**Figure 1:**
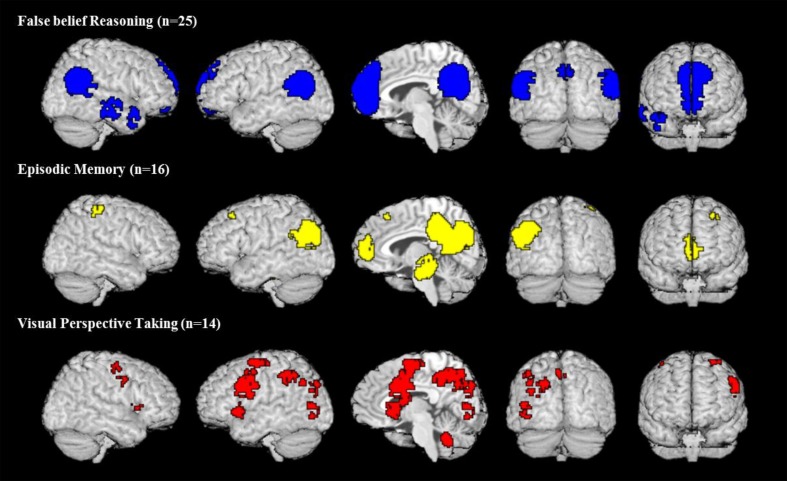
**Activation maps of meta-analyses for three different domains**. All maps are thresholded at voxel-wise threshold of *p* < 0.005 uncorrected and a cluster extent threshold of 10 voxels. Activations of all meta-analyses are superimposed on the Talairach template.

**Figure 2:**
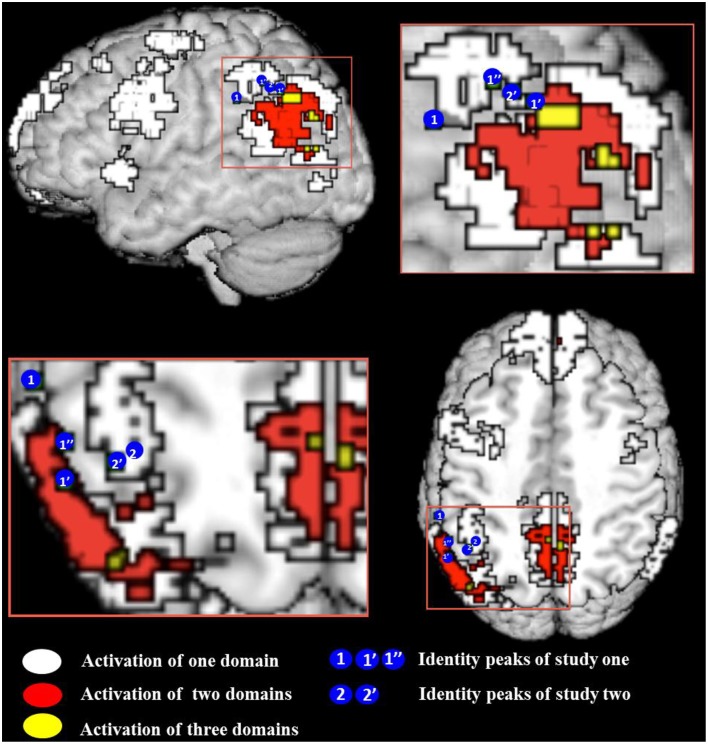
**Conjunction map of all meta-analyses false belief (FB), episodic memory (EM), and visual perspective taking (vPT)**. White indicates the regions activated by one meta-analysis, red and yellow indicate the conjunction of at least two and three meta-analyses. Location of activation peaks for the identity contrast are shown as blue circles with the number of the study—see **Table 4** for peak coordinates and overlap details (areas of the blue circles do not reflect the actual size of the activation). All meta-analytic maps were thresholded at voxel-wise threshold of *p* < 0.005 uncorrected and a cluster extent threshold of 10 voxels. Activations of all meta-analyses are superimposed on the Talairach template.

All meta-analytic maps were thresholded at a voxel-wise threshold of *p* < 0.005 uncorrected and a cluster extent threshold at 10 voxels. Figure 1 shows the activation maps for each meta-analysis. As one can see there is a potential overlap among all three kinds of tasks only on the left lateral hemisphere (2nd and 4th column) in the parietal lobe and medially (3rd column) in the posterior parts around the precuneus. Figure 2 shows these two areas in detail. Overlap in Figure 2 was determined by conjunction analysis between maps of significant meta-analytic activation (i.e., conjunction determined areas significantly activated in map1 AND map 2). This was done with the image calculator in SPM8 (www.fil.ion.ucl.ac.uk/spm/).

The observed pattern of overlap among activations from the three meta-analyses conforms to the view by Cabeza et al. (2012) that the IPL and possibly also parts of the anterior (y close to −60) precuneus have the overarching function of tracking perspective: All three kinds of tasks overlap in a central area but also activate individually surrounding areas. We can now use the activations shown in the meta-analyses to check whether other perspective tasks, which were tested only in a few studies, overlap with the meta-analysis. Since activations in individual studies tend to be variable we cannot expect each single study to show overlap with the central area where the three meta-analytic activations overlap. Hence our criterion for supporting evidence is that the activation of perspective tasks from individual studies must overlap with at least one of the activation areas of the meta-analysis.

As a first test case we have two studies that used false sign vignettes (Perner et al., 2006; Aichhorn et al., 2009). They looked at the regions of interest defined by the false belief vs. photo vignettes (Saxe and Kanwisher, 2003). In both studies the false sign vignettes activated the right IPL less than the false belief with no difference to the photo vignettes. In the left IPL the vignettes activated more strongly than the photo with no difference to the false belief. The same held true for the precuneus as expected under the regional specificity hypothesis that perspective tasks like the false sign task should overlap with other perspective tasks in the left IPL and precuneus.

Moreover, the left IPL was also reported in studies using conceptual perspective tasks (Goel et al., 1995; Ruby and Decety, 2003). Goel et al. (1995) asked participants to describe how, e.g., a person like Columbus from the perspective of the 15th century could infer the function of a modern artifact, e.g., hair drier. They reported activation in the left IPL and precuneus. Ruby and Decety (2003) asked medical students to respond to health-related questions either from their own perspective or from the perspective of a “lay person.” Third person vs. first person activated the IPL/TPJ on the left and also on the right (to be expected since the third person perspective relied heavily on what the lay person believes about the issues). No precuneus activation was reported. So these studies confirm that the left IPL and (with less certainty) precuneus have the overarching function of tracking perspective.

In the following we test the prediction. We argue that processing identity statements requires the tracking of perspectives and thus should activate these areas in the left IPL and in precuneus whose overarching function is to track perspective.

## Study 2: identity 1

We want to provide a new test of the regional specificity hypothesis that the left IPL and possibly the anterior precuneus have the overarching function of tracking perspectives. For this test we try to identify an unusual candidate for a perspective task and then investigate whether it, too, activates the predicted areas. For our test we focus on identity statements, which on first blush seem to have little affinity to perspective. However, identity statements, e.g., “the driver is the tour guide” involve different labels (“driver,” “tour guide”) for the same individual. Psycholinguists often say that identifying an object under different labels puts a different perspective on that object (see Clark, 1997; Tomasello, 1999). Frege's (1967) and May's (2001) famous analysis of identity statements brings out the importance of perspective in the form of modes of presentation. In the identity statement “the driver is the tour guide” the expressions “the driver” and “the tour guide” refer to the same individual (person X). If the meaning of these expressions were understood only in terms of their referent (person X) then the identity statement would not be informative, for it would reduce to “person X is person X.” The statement only makes sense if one is sensitive to the fact that each constituent expression provides a different mode of presentation (sense or perspective) of that particular individual to which they both refer.

Mental files (Perry, 2002; Recanati, 2012) provide a helpful alternative approach for seeing how perspective enters identity statements and why they have an affinity to understanding belief (Perner and Leahy, 2015). Use of the referential expressions “the driver” and “the tour guide” in discourse create two mental files for the same referent. They capture the two ways how one conceives of person X. The files contain the information that one has accumulated for the person under each conception. The identity statement makes clear that these are but different conceptions of a single person. One can then either keep the two files separate but link them (Perry, 2002) or merge them into a single file for person X^5^. Similarly when representing what someone mistakenly thinks, e.g., Julia in the false belief vignettes about the ice cream van, two mental files are created, a regular file registering what one knows about the van, and a vicarious file indexed to Julia. The vicarious file is linked to the regular file (Recanati, 2012) to represent sameness of referent, and on the file one registers what Julia thinks about the van. In other words, the regular file captures how oneself conceives of the van and the vicarious file how Julia conceives of it. Both, understanding identity statements and attributing false beliefs, require linked files for a single referent. This common requirement can explain why understanding identity and belief emerges at the same age (Perner et al., 2011; Perner and Leahy, 2015).

If one wants to assess brain activation due to identity statements, one has to make sure that the stimulus material induces the relevant processing. There is a danger that listeners to a statement like, “the driver is the tour guide,” do not—as intended—think of two individuals, the driver and the tour guide, and then understand that there is but a single individual who is the driver and the tour guide. Instead, especially under the repetitive presentation conditions typical for fMRI, participants may gloss the sentence as “the driver is a tour guide,” i.e., they only ever think of one individual as driver and then encode that he works as a tour guide. This would ruin our identity condition.

Therefore, we took care that participants naturally thought of two different individuals before they were given the critical identity information, e.g.:

S1: “On this bus trip the tour guide talks to the passengers as much as the driver.”

The listener now thinks of two people, the tour guide and the driver. Then the identity statement is given:

S2: (+IDENT): “The tour guide is also the driver.”

This informs the listener that there are not two people involved but only one person. This should—according to our Fregean analysis—make the listener aware that “tour guide” and “driver” are just two different perspectives (modes of presentation, conceptions) of that one person. A suitable control statement needs to be syntactically and in other aspects as similar as possible to our critical statement without involving an identity relation, e.g.:

S2: (−IDENT): “The tour guide has an assistant^6^.”

Unfortunately, in addition to the minor linguistic differences, there is another not so negligible difference between these two versions of sentence S2 to contend with. When two different referential expressions like “the tour guide” and “the driver” are used we naturally think (build a mental model) of two distinct people. Although natural, it is strictly speaking a rash interpretation, as the ensuing identity information makes clear. There are not two but only one person talked about. In other words, the listener has to revise her rashly formed belief of two distinct people on this bus trip to believing that there is only one person filling both positions. Quite plausibly the listener will also notice that she has been briefly misled, which amounts to attributing a false belief to herself in the immediate past. So we need to control for this in order to prevent misinterpreting activations due to the listener attributing a false belief to herself as activations caused by identity statements. In order to control for this possibility we introduced two further variations of sentence S2 one involving belief revision without any identity information:

S2: (+REVISION): “Today, the tour guide talks more than the driver.”

This would also lead to revision of the belief created by the first sentence that both people always talk the same amount. In contrast to S2 (+IDENT) it does not involve an identity statement. In order to identify activations due to this belief revision we also used a control that was syntactically similar to S2 (+REVISION) without involving a belief revision. It just adds more information:

S2: (−REVISION): “The tour guide also earns as much as the driver.”

The objective of our study is to see whether the identity contrast (+IDENT > −IDENT contrast) activates identifiable regions of the brain. The most general question (1) is whether there is any such region. More specifically (2) we expect activations in areas relevant for perspective awareness, specifically the network in the left IPL identified in Figures 1, 2 by meta-analyses of other perspective tasks.

However, these expectations have to be modulated by results of our belief revision control contrast (+REVISION > −REVISION), which indicates that belief revision leads to self-attribution of a false belief. In this case the identity contrast (+IDENT > −IDENT) can only be interpreted outside these regions unless the (+IDENT > +REVISION) contrast is also significant, i.e., the identity statement activates the region in addition to any false belief attribution caused by belief revision^7^.

## Method

### Participants

Twenty-one university students (6 males, mean age 23.95 years, *SD* = 3.96) participated in this study for course credits and small monetary reimbursement. All participants were native German speakers, had normal or corrected-to-normal vision, and had no history of neurological disorder. A written informed consent was obtained from all the participants before scanning. The ethics committee of the University of Salzburg approved the study.

### Stimuli

The stimuli consisted of written German sentences (example sentences translated in English are presented in Table 1). During the whole experiment, 18 different scenarios were used to administer the four conditions of interest (+IDENT, −IDENT, +REVISION, –REVISION). For a particular scenario there was a standard first sentence S1. The second sentence (S2) differed for each of the four conditions. This yielded 72 different vignettes. The whole scanning session was split into three runs consisting of six trials of each condition. To avoid sequence effects vignettes derived from the same scenario were never presented near each other. Moreover, participants were instructed that all vignettes could be treated as independent and nothing had to be remembered for longer than one trial. Thirty percent of the vignettes were followed by a control question. Whether the question was about the first or the second sentence, the side of “Yes” and “No” response, and the side of the correct answer-key was randomized. Stimulus presentation, timings and response recording were controlled by Presentation software (Neurobehavioral System, Albany, CA, USA).

**Table 1:** **Example sentences of Study 2 (translated from German; see Table S2. in supplementary material for more original examples in German)**.

**Conditions**	**Context sentence (S1) 5 s**	**Condition sentence (S2) 6 s**	**Verification sentence 6 s**
Identity (+IDENT)	On this bus trip the tour guide talks to the passengers as much as the driver^a^	The tour guide is also the driver	Thus, a tour guide is on the bus. <yes>
No Identity (−IDENT)		The tour guide has an assistant	Thus, the assistant always comes along. <yes>
Belief Revision (+REVISION)		Today, the tour guide talks more than the driver	Thus, today one of them does more of the talking. <yes>
No Belief Revision (−REVISION)		The tour guide also earns as much as the driver	Thus, both earn different amounts of money. <no>

a *The same context sentence was used for all conditions*.

### Procedure and design

Participants were asked to read short vignettes. Every trial consisted of at least two sentences. At the beginning only the first sentence S1 (e.g., “On the bus trip the tour guide talks as much as the driver”) was presented for 5 s. Then the second sentence S2 (e.g., “The tour guide is the driver”) was added and both sentences remained for a further 6 s on the screen. In 70% of the trials of each scanning run the vignette was followed by the word “CONTINUE” (500 ms) to indicate that the trial had finished and the next one was about to start. To ensure the compliance of participants, they had to answer in the remaining trials a simple question within 6 s (e.g., “Thus a driver is on the trip: Yes?/No?) by pressing a key. Between trials a fixation cross was presented with varying duration, ranging from one to 4 s. Correct affirmative and negative answers were balanced within conditions.

The no-question trials lasted for an average of 14 s and question trials for an average of 19.5 s. Before the start of each trial there was an inter-stimulus interval of 1–4 s. The sequence of the trial and the inter-stimulus interval was optimized using Russ Poldrack's script (we optimized a fixed time span for four conditions of interest and one rest condition; http://sourceforge.net/projects/fmri-toolbox/files/optimize_design/1.1/).

## fMRI data acquisition

Functional and structural imaging was acquired with a Siemens 3 Tesla Tim-Trio Scanner, located at Christian-Doppler-Clinic, Salzburg. Functional images sensitive to the BOLD contrast were obtained with a T2^*^-weighted gradient echo-planar imaging (EPI) sequence using a 32 channel head coil. Per subject, three sessions, and a total of 239 EPI images including 6 dummy scans at the beginning of the functional images were scanned to allow transient signals to diminish (*TR* = 2000 ms; *TE* = 30 ms; matrix size = 96 × 96; voxel size = 2.187 × 2.187 × 3.58 mm^3^; slice thickness = 3.0 mm; slice gap 0.6 mm; FOV = 210 mm; flip angle = 70°). Thirty-six axial slices were acquired in descending order parallel to the bicommissural (co-planar with AC–PC) line along the z-axis. In addition to functional scanning, sagittally oriented high-resolution structural scan was acquired (T1-weighted MP-RAGE sequence; *TR* = 6.73 ms; *TE* = 3.14 ms; voxel size 0.797 × 0.797 × 1.2 mm^3^; slice-thickness = 1.2 mm; matrix 256 × 256; FOV = 204 mm; 170 slices per volume; flip angle = 8°).

## fMRI data processing

Preprocessing and statistical data analysis was performed by Statistical Parametric Mapping (SPM8, http://www.fil.ion.ucl.ac.uk/spm), implemented in MATLAB 7.3 [R2006b] (Matworks, Sherborn, MA) runtime environment. Images were slice-time and motion corrected by standard SPM8 algorithms. Functional images were registered to the SPM8 EPI template. The structural scan was co-registered onto the mean functional images of each session and segmented. Segmentation parameters were used for normalization of structural and functional images to MNI space (Montreal Neurological Institute, McGill, Montreal, Canada) template. The normalized images were resampled to isotropic 3 × 3 × 3 mm voxels and smoothed with an 8 mm full width at half maximum (FWHM) Gaussian kernel.

The preprocessed data were analyzed using a general linear model (GLM) approach. The functional data were high-pass filtered in order to remove frequencies below 1/128 Hz to reduce low frequency drift. The serial correlation was taken into account using the autocorrelation AR (1) model, as implemented in SPM8. On individual level contrast the four conditions relative to fixation baseline were modeled. The condition sentence (S2) was modeled as an event of interest for all four conditions separately. The context sentence (S1) and the verification questions were modeled as regressors of no interest. Additionally, realignment parameters and session mean were included as covariates. The first level contrast images of each subject were used for the second level (random effects) analysis, that allows for the generalization to the population. The statistical comparisons were inspected at a voxelwise threshold of *p* < 0.001 together with a cluster extent threshold of *p* < 0.05, corrected for family-wise error (FWE).

## Results

### Behavioral results

The overall accuracy was around 90% (see Table 2), indicating that the participants were attentive and understood the task. We computed a One-Way repeated measure ANOVA using participants' hit-rates. There was no statistically significant difference in accuracy across the four conditions [*F*_(3, 60)_ = 1.488, *p* = 0.22, η^2^ = 0.069]. This implies that the difficulty level was similar across all conditions.

**Table 2:** **Behavioral results of Study 2: mean accuracy in percent hit rate (SD)**.

	**Conditions**			
	**+IDENT**	**−IDENT**	**+REVISION**	**−REVISION**
Hit-Rate (%) *SD*	91.3 (14.3)	87.2 (8.5)	90.3 (12.4)	94.3 (9.1)

We will not report reaction times (RT) for the sake of brevity. This is because RTs were collected on the Yes/No responses to the questions presented within the response window of 6 s, they do not reflect the actual time taken to comprehend the vignettes but rather the time taken to read the question and respond “yes” or “no” to the visual cue.

### Neuro-imaging results

We report all regions for identity and belief revision contrasts at FWE cluster level corrected *p* < 0.05 in Table 3.

**Table 3:** **Supra-threshold whole brain activation of identity and belief revision in Study 2**.

**Region**	**H**	***k***	**Max *Z***	**MNI coordinates**		
				***x***	***y***	***z***
**IDENTITY: +IDENT > −IDENT**						
Supramarginal Gyrus (PF L)	L	90	4.46	−60	−34	37
*Angular Gyrus (PFm L)*	*L*	*–*	*3.84*	*−54*	*−52*	*43*
*Supramarginal Gyrus (PF L)*	*L*	*–*	*3.55*	*−54*	*−43*	*46*
**BELIEF REVISION: +REVISION > −REVISION**						
Angular/Lateral occipital cortex, superior division (Pga L)	L	136	4.53	−51	−64	34
*Angular Gyrus (PFm L)*	*L*	*–*	*4.26*	*−42*	*−58*	*31*
Middle Frontal Gyrus (BA6 L)	L	95	4.43	−39	11	52
*Middle Frontal Gyrus (BA44 L)*	*L*	*–*	*3.65*	*−39*	*17*	*40*
*Middle Frontal Gyrus (BA44 L)*	*L*	*–*	*4.03*	*−42*	*20*	*49*

*Significant cluster are reported at p < 0.05 FWE cluster level corrected*.

*Regions are reported from posterior to anterior. Regions, Anatomical labeling corresponding to the cluster peak and sub-peak (according to Harvard-Oxford cortical and subcortical structural atlases). Regions in brackets, Anatomical labeling corresponding to the cluster peak and to sub-peaks are also reported according to Jülich histological cyto-and myelo-architectonic atlas by Eickhoff et al. (2005, 2006, 2007); H, Hemisphere of peak; k, cluster extent in voxel; Max Z, Maximum Z-value; sub-peaks of the regions with cluster level below p < 0.05 FWE corrected are reported in italics*.

Of main interest was the identity contrast comparing identity with its control condition (+IDENT > −IDENT). Only one parietal activation in the left inferior parietal lobe (left IPL) with its main peak and one of the sub-peaks in the supramarginal gyrus (SMG) and another sub-peak in angular gyrus (AG) was FWE cluster level corrected significant at *p* < 0.05. Comparison in the opposite direction (−IDENT > +IDENT) did not reveal any significant cluster.

The belief revision contrast (+REVISION > −REVISION) activated two clusters FWE corrected at *p* < 0.05; one in the left IPL (angular gyrus) and the other in the left middle frontal gyrus. The inverse contrast (−REVISION > +REVISION) did not show any significant activation. For each relevant contrast, overlap with meta-analytic activations was tested in the following way: Based on the peak-voxel coordinate activated in this contrast, we checked for each meta-analysis map if significant activation was found here. The left angular gyrus cluster peak and sub-peaks of the belief revision contrast were also significantly activated in our false belief and in our episodic memory meta-analysis. This overlap suggests that by becoming aware of having to revise one's belief one attributes a false belief to oneself ^8^.

### Identity and belief revision

**Figure 3:**
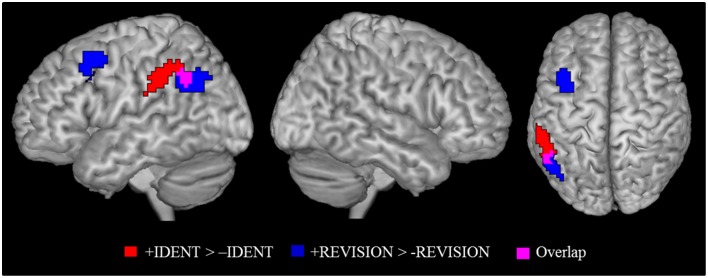
**Identity contrast (red); belief revision contrast (blue), and overlap between the two contrasts (magenta)**. Activation cluster are superimposed on an MNI template. All contrasts were shown at *p* < 0.05 FWE cluster level corrected threshold.

As argued earlier in the explanation of our experimental design, we needed to check if brain activity for identity statements can also be found for other statements that cause belief revision. This is necessary in order to not misinterpret activations as caused by identity statements when in fact they may be due to the listener attributing a false belief to herself. Figure 3 shows the activation patterns for the identity contrast (+IDENT > −IDENT) and for belief revision contrast (+REVISION > −REVISION). Overlap was determined by inclusively masking the belief revision contrast with the identity contrast (at the default threshold of *p* < 0.001). We found overlap in the left angular gyrus (−54, −52, 43) *k* = 15 and in the right lateral occipital cortex (48, −64, 40) *k* = 5. Given this overlap, we cannot rule out that our identity statements were activating these left IPL areas because they caused a belief revision. Therefore, to detect areas activated by the identity contrast independently of belief revision, we removed (exclusively masked) all regions activated by belief revision (*p* < 0.001 uncorrected) from the identity contrast. The identity contrast outside the belief revision mask stayed significant in the left IPL, (*k* = 74) at FWE cluster level corrected *p* < 0.05 with the cluster peak (−60, −34, 37) and two sub-peaks in the left supramarginal gyrus (−54, −43, 46; −54, −49, 43).

This result confirms our expectation based on developmental data that identity statements activate the left left IPL, as the region is sensitive to perspective differences. To answer our more specific question, whether identity statements activate a more specific “perspective region” in the left IPL, we need to define a region of interest. Here we adopt the *overarching view* of Cabeza et al. (2012) that allows for subdivisions within a broad brain region. The broad region (in our case, the left IPL) has a global function (representing perspective differences) and its various sub-regions mediate different aspects (false beliefs, visual perspectives, etc.) of the global function. The expected pattern of finding is that each perspective task should activate the broad region and partially overlap with activations by the other tasks. For this purpose we used the results from our meta-analysis. We checked for each peak voxel if a meta-analysis showed significant activation at the given coordinate. Results of this examination are given in Table 4. Figure 2 show the overlay of identity contrast peaks with the activations shown in the meta-analyses.

**Table 4:** **Overlap (+) of identity activations in Study 2 and 3 with false belief, episodic memory, and visual perspective taking**.

	**Peak label**	**Overlap with**					
		**MNI coordinates**			**FB**	**EM**	**vPT**
**STUDY 2**							
Cluster peak: SMG	1	−60	−34	37	–	–	–
Sub-peak: AG	1′	−54	−52	43	+	–	–
Sub-peak: SMG	1^”^	−54	−43	46	–	–	–
**STUDY 3**							
Cluster peak: SMG	2	−39	−46	43	–	–	+
Sub-peak: SMG	2′	−42	−49	46	–	–	+

*Peak label. corresponds to the labeling in Figure 2. MNI peak coordinates of Study 2 and Study 3 were converted into Talairach space to have the same stereotactic space as the meta-analysis. FB, False belief reasoning; EM, Episodic memory; vPT, Visual perspective taking*.

We were unable to directly compare results because our imaging studies and the meta-analyses were analyzed in different coordinate systems. All meta-analyses had to be performed in Talairach space, as the default coordinate system of Effect-Size Signed Differential Mapping (ES-SDM) software, version 2.31 for meta-analysis (Radua et al., 2010, 2012); http://www.sdmproject.com), while our data were normalized in MNI space. We thus converted our left SMG cluster peak and sub-peaks into Talairach space (see Table 4). We constructed a 3 mm in diameter sphere—which corresponds to the voxel-size of our images –around those peaks using the WFU PickAtlas (http://fmri.wfubmc.edu/software/PickAtlas). One of the sub-peak spheres in the angular gyrus that overlapped with belief revision also overlapped significantly with false belief meta-analysis areas [a coordinate-wise search for foci that were significantly activated in both analyses, performed in MRIcron (http://www.mccauslandcenter.sc.edu/mricro/mricron/)]. This confirms our prediction that the processing of identity statements might have led participants to correct their rashly formed belief about the “tour guide” and the “driver” as being two distinct people to believing that there is only one person filling both positions.

## Discussion

Our initially formulated expectations for the identity contrast received a fairly clear answer. (1) We were able to identify at least one region that is significantly (FWE-corrected) activated by the identity contrast. (2) This identity cluster lies in the left IPL as predicted in the hypothesis; tasks that require awareness of perspective will activate this region. (3) Although the main peak and one of the sub-peaks of the identity cluster were in the left supramarginal gyrus another sub-peak was in the angular gyrus that overlapped with false belief activation of the meta-analysis.

This pattern of results fits the overarching view (Cabeza et al., 2012) that the left IPL has the overarching function of registering (actual or potential) perspective differences. Different tasks modulate this function, showing activation in different parts of the IPL but such that they partially overlap, as the meta-analysis of perspective tasks (false belief, visual perspective taking, and episodic memory) show. Our results extend this picture to identity tasks.

Overlap of the identity contrast in our study happened to occur in the meta-analytic areas for false belief activation. One problem of interpretation occurred because our identity task involved belief revision. Belief revision, as we were able to show, also activates in the meta-analytic false belief area, suggesting that belief revision, at least when one is aware of it, amounts to attributing a past false belief to oneself. This raises the possibility that the overlap between the identity contrast and false belief may be due to the belief attribution caused by the belief revision inherent in our identity condition. Therefore, it would be reassuring if overlap with perspective tasks can be found without the involvement of belief revision in identity tasks. This was investigated in the next study.

## Study 3: identity 2

The objective of this experiment is to check whether the central results of Study 2 can be replicated by avoiding the confounding of identity statements with belief revision. The confound resulted from our decision to prevent participants glossing a simple identity statement like, “the mayor is the lawyer,” as an attributive statement, “the mayor is a lawyer.” While the former mentions two people (the mayor and the lawyer) and then says something about their identity, the latter only mentions one person (the mayor) and then informs about that person's profession. To avoid such a gloss we used a context sentence to establish the mayor and the lawyer as two different individuals in participants' minds. With the identity statement participants then learned that mayor and lawyer are the same person. This led inevitably to a belief revision.

For this current experiment we decided to run the risk of participants glossing some of the identity statements as attributive assertions. If this results in similar activations as in Study 2 (especially of the left IPL) we can conclude that these activations are not due to belief revision. Trying to minimize the risk of an attributive gloss, each statement used a common description (the lawyer) as its first referential term and as the second term a proper name (Mr Müller). Although one can easily gloss “Mr Müller is the lawyer” as “Mr Müller is a lawyer),” it is harder to do so with “The lawyer is Mr Müller^9^.”

## Method

### Participants

Seventeen (5 males; mean age 24.6 years, *SD* = 4.9 years) right-handed university students participated in this study for course credits and small monetary reimbursement. All participants were native German speakers, had normal or corrected-to-normal vision, and had no history of neurological disorder. A written informed consent was obtained from all the participants before scanning. The ethical committee of the University of Salzburg approved the study.

### Design and stimuli

The study had five conditions (see Table 5) consisting of written German sentences. Three context conditions were introduced with a context sentence mentioning two people, e.g., a doctor and a lawyer. In the *identity-with-context* (IDENTc) condition an identity statement followed which expressed that one of these people (lawyer) was identical to, e.g., Mr. Müller. In the *predication-with-context* (PREDc) condition the second sentence predicated some attribute of, e.g., the lawyer. In the *context-only* (C) condition this second sentence was omitted. This condition served as a parameter of no interest for comparing IDENTc with PREDc. Two additional conditions served to replicate a finding of a pilot study using simple identity statements without any background context (*identity only*, IDENTo). The pilot activation was difficult to interpret, as the design didn't have any explicit low-level baseline. We therefore included, a low-level baseline condition (BL) with simple sentences (e.g., the glasses are old-fashioned).

**Table 5:** **Example sentences of Study 3 (translated from German; see Table S3 in supplementary material for more original examples in German)**.

**Conditions**	**Context sentence (S1) 4.5 s**	**Condition sentence (S2) 3 s**	**Comprehension questions? 5.5 s**
Identity-with-context (IDENTc)	The doctor saves the lawyer after the accident	The lawyer is Mr. Moser	Who is Mr. Moser?^a^
Predication-with-context (PREDc)		The lawyer is young	Who is young?
Context only (C)		–	Who saved the lawyer?
Identity only (IDENTo)	–	The neurologist is Dr. Phillips	Who is the neurologist?
Baseline (BL)		The chair is old-fashioned	What is old-fashioned?

a *The comprehension question in conditions with context sentence varied accordingly (see design and stimuli section of Study 3 for details)*.

Twenty-seven different sentences were used per condition, resulting in a total of 135 trials in the experiment. All sentences of IDENTc and PREDc conditions were formed by linking a referential noun phrase, e.g., “The lawyer” by the particle “is” with either a proper name to form an identity statement or with an adjective to form predicative sentences. The noun phrases were counterbalanced for the two conditions.

We controlled for sentence length in all conditions. The mean number of letters in the context sentences (S1) varied between conditions from 40.7 (±6.0) in IDENTc to 40.5 (±6.0) in PREDc to 41.2 (±7.0) in C, and the average letter count in the identity sentences (S2) varied from 22.19 (±2.6) in IDENTo to 23.5 (±2.9) in IDENTc. There was no significant difference across conditions for context or for identity sentences (all *p*'s ≥ 0.35).

The presentation times for sentences S1 and S2 are shown in Table 5. On 30% of trials a comprehension question was asked. In the context conditions this question could be about any of the three names mentioned (for example see Table 5: “Who saved the lawyer?” or “Who did the doctor save?” or “Who is Mr. Moser?”). This variation was to ensure that participants had to integrate sentences S1 and S2 in a single model. In the conditions without context the question only varied between the two names that referred to the same individual (e.g., “Who is Mr. Moser?” or “Who is the neurologist?”). The total time provided was 5500 ms: the question was presented for 3000 ms, followed by 1000 ms of black screen, and finally the answer option for 250 ms (e.g., <the lawyer> <the doctor>). Correct and incorrect options to the question were balanced across conditions to avoid confounds of any strategies to answer the questions and habitual finger use. Stimulus presentation, timings and response recording were controlled by the Presentation Software (Neurobehavioral System, Albany, CA, USA).

Functional neuroimaging was divided into three sessions. Each session comprised 45 trials, 9 pre-condition trials and 14 comprehension questions. The order of the presentation of sessions was counterbalanced across participants. A single trial without question lasted for 11 s in the conditions with context, 6.5 s in the identity only and in the baseline condition, and 8 s in the context only trials. Each single session lasted for 10.35 min, and the whole functional scanning of the experiment took 31.07 min.

### Procedure

The participants were given a training session before the start of the scanning. They were specifically instructed to read and understand the sentences carefully, and that they would sometimes be asked to answer a question to verify their attention and comprehension of the vignettes. Behavioral responses were collected using an MRI-compatible response box.

### fMRI data acquisition

Functional and structural imaging was acquired with a Siemens 3 Tesla Tim-Trio Scanner, located at the Christian-Doppler-Clinic, Salzburg. Functional images sensitive to the BOLD contrast were obtained with a T2^*^-weighted gradient EPI sequence using a 32 channel head coil. Per subject, three sessions, a total of 260 EPI images including 6 dummy scans at the beginning of the functional images were scanned to allow transient signals to diminish (*TR* = 2250 ms; *TE* = 30 ms; matrix size = 64 × 64; voxel size = 3.0 × 3.0 × 3.0 mm^3^; slice thickness = 3.0 mm; slice gap 0.3 mm; *FOV* = 192 mm; flip angle = 70°). Thirty-six axial slices were acquired in descending order parallel to the bicommissural (co-planar with AC–PC) line along the z-axis. In addition for each subject sagittally oriented high-resolution structural scan was acquired (T1-weighted MP-RAGE sequence; *TR* = 2300 ms; *TE* = 2.91 ms; voxel size 1.0 × 1.0 × 1.0 mm^3^; slice-thickness = 1.00 mm; matrix 256 × 256; *FOV* = 256 mm; 192 slices per volume; flip angle = 9°).

### fMRI data processing

Preprocessing and statistical data analysis was performed using Statistical Parametric Mapping (SPM8, http://www.fil.ion.ucl.ac.uk/spm), implemented in MATLAB 7.6.0.324 [R2008a] (Matworks, Sherborn, MA) runtime environment. Images were slice-time and motion corrected by standard SPM8 algorithms. Functional images were registered to the SPM8 EPI template. The structural scan was co-registered onto the mean functional images of each session and segmented. The structural and functional images were normalized to MNI (Montreal Neurological Institute, McGill, Montreal, Canada) template. The normalized images were resampled to isotropic 3 × 3 × 3 mm voxels and smoothed with an 8 mm full width at half maximum (FWHM) Gaussian kernel.

The preprocessed data were analyzed using a GLM approach. Per subject, and session, IDENTc, PREDc, IDENTo, and BL condition sentence (S2) was modeled as a separate regressor of interest with the duration of 3 s and convolved with the hemodynamic response function. The S1 of conditions with context (IDENTc and PREDc) and C were modeled with the duration of 4.5 s as a single regressor of no interest. We also modeled the comprehension question with the duration of 5.5 s as a separate regressor of no interest. Additionally, realignment parameters and session means were included in the design matrix as covariate. The low frequency noise was removed by high-pass filter with a cut-off of 128 s, and serial correlation was taken into account using an autocorrelation AR (1) model, as implemented in SPM8. At the individual level of contrasts the four conditions were modeled separately relative to an implicit baseline.

Data at the second level were subject to a random effects analysis to allow for population inference. We computed paired *t*-tests between contrasts of interest. Whole brain results are reported at a voxel-wise threshold of *p* < 0.001 together with a FWE cluster level corrected threshold of *p* < 0.05.

## Results and discussion

### Behavioral results

Overall accuracy was very high 97.51% (see Table 6), with an overall miss rate of 5.06%. The high accuracy was a good indicator that participants were attentive and understood the task. Given that accuracy was at ceiling in this study, it was unnecessary to carry out statistical tests here.

**Table 6:** **Behavioral results of Study 3: mean accuracy in percent hit rate (SD)**.

	**Conditions**				
	**IDENTc**	**PREDc**	**IDENTo**	**BL**	**C**
Hit-Rate (%) SD	98.4 (4.6)	97.4 (5.9)	97.6 (5.5)	100 (0)	94.1 (5.7)

We do not report reaction time (RT), since the RTs depended on the time spent to answer the comprehension question they do not reliably reflect the actual time taken to comprehend the vignettes.

### Neuro-imaging results

The main contrast of interest is the one between *identity-with-context* and *predication-with-context* (IDENTc > PREDc). The whole brain analysis for this contrast showed two significant FWE-corrected clusters (see Table 7). One cluster lies in the precuneus on the left side, the other, in the left supramarginal gyrus as predicted.

**Table 7:** **Supra-threshold whole brain activation of identity vs. predication in context conditions of Study 3**.

**Region**	**H**	***k***	**Max *Z***	**MNI coordinates**		
				***x***	***y***	***z***
**IDENTITY: IDENTc > PREDc**						
Precuneus Cortex (7M L)	L	88	4.07	−12	−67	28
*Lateral Occipital Cortex, superior*	*L*	*–*	*3.61*	*−15*	*−76*	*46*
*division (7P L)*						
*Precuneus Cortex*	*L*	*–*	*3.58*	*−18*	*−70*	*22*
Supramarginal Gyrus (hlP1)	L	67	4.09	−39	−46	43
*Supramarginal Gyrus (hlP1)*	*L*	*–*	*4.06*	*−42*	*−49*	*46*
**INVERSE IDENTITY: PREDc > IDENTc**						
Temporal Pole (No label)	R	146	4.90	48	8	−26
*Superior Temporal Gyrus (No label)*	*R*	*–*	*4.24*	*57*	*−10*	*−8*
*Superior Temporal Gyrus (No label)*	*R*	*–*	*4.58*	*54*	*2*	*−17*
Temporal Pole (No label)	L	191	5.69	−51	11	−20
*Superior Temporal Gyrus (No label)*	*L*	*–*	*4.12*	*−51*	*−4*	*−17*
*Temporal Pole (No label)*	*L*	*–*	*5.19*	*−45*	*14*	*−26*

*Significant cluster are reported at p < 0.05 FWE cluster level corrected*.

*Regions are reported from posterior to anterior. Regions, Anatomical labeling corresponding to the cluster peak and sub-peak (according to Harvard-Oxford cortical and subcortical structural atlases). Regions in brackets, Anatomical labeling corresponding to the cluster peak and sub-peak are also reported according to Jülich histological cyto-and myelo-architectonic atlas by Eickhoff et al. (2005, 2006, 2007); H, Hemisphere of peak; k, cluster extent in voxel; Max Z, Maximum Z-value; sub-peaks of the regions with cluster level below p < 0.05 FWE corrected are reported in italics*.

**Figure 4:**
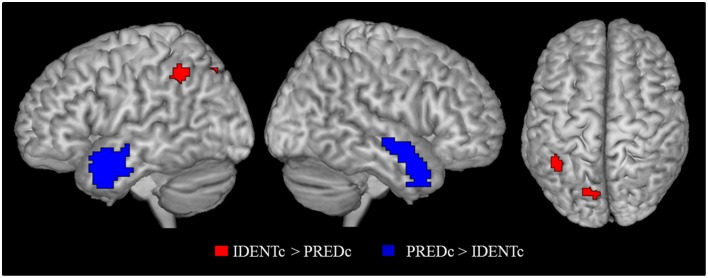
**Contrast of identity-with-context > predication-with-context (red) and the inverse contrast (blue)**. Activation cluster are superimposed on an MNI template. All contrasts were shown at *p* < 0.05 FWE cluster level corrected threshold.

The inverse contrast (PREDc > IDENTc) showed activations in quite distant parts of the brain (see Table 7 and Figure 4). Two large FWE corrected clusters were located in the left and right temporal pole area associated with social scripts and social concepts (Zahn et al., 2007; Ross and Olson, 2010) and prevalent in theory of mind studies (Schurz et al., 2014). This is plausibly due to the fact that predicative information about a person (the lawyer is young) stimulates social thoughts more strongly than a statement that this person is identical to someone (Mr. Moser) about whom one has no information.

The identity statement without context compared to the baseline condition (IDENTo > BL) showed significant activation of the left supplementary motor area (SMA), left precentral gyrus, left lateral occipital cortex, bilateral cerebellum, left inferior frontal gyrus (IFG) and right superior parietal lobe activation at FWE cluster level corrected at *p* < 0.05^10^.

### Relation to study 2 and meta-analysis

The predicted activation by the identity contrast (IDENTc > PREDc) in Study 3 was in close vicinity to the activation observed in the left IPL for the identity contrast (+IDENT > − IDENT) in Study 2. After masking the belief revision clusters the average Euclidian distance between the sub-peaks of Study 2 (−54, −43, 46, and −54, −49, 43) and the cluster peak and sub-peak (−39, −46, 43, and −42, −49, 46) of Study 3 was 14.16 mm. In order to assess the support for the claim that all perspective tasks activate the overarching region in the left IPL we tested for overlap of the identity contrast (IDENTc > PREDc) with areas shown in the meta-analyses. Using the same method as in Study 2 we converted the left SMG cluster peak and sub-peaks of Study 3 (see Table 4, Figure 2 for overlap details) into Talairach space, and constructed a 3 mm in diameter sphere around it, which overlapped with the regions of the visual perspective taking meta-analysis and bordered on the areas activated by false belief vignettes and episodic memory.

The fact that the activations found in the two identity studies did not directly overlap is mitigated by the strong connectivity between the subareas of the IPL in which the activations occurred. The SMG cluster peak (−60, −34, 37) and the sub-peaks (−54, −52, 43, and −54, −43, 46) of the identity contrast in Study 2 (see Table 3) fall into the cytoarchitectonic area of the left PF and PFm region (Caspers et al., 2006, Jülich Histological Atlas). The SMG cluster peak (−39, −46, 43) and sub-peak (−42, −49, 46) of Study 3 (see Table 7) are located in the left intraparietal sulcus (subregion: hIP1; Choi et al., 2006; Jülich Histological Atlas). According to Caspers et al. (2011) structural connectivity fingerprints show a strong connection between PF, PFm, and hIP1 region. The strong connectivity among the different areas activated by our studies supports the conclusion that different activation points reflect activity of an overarching functionally related network.

## General discussion

### Main achievements

Our studies produced two main achievements. (a) We were able to establish that the ability to track perspective, which marks an important advance in child development around 4 years of age, manifests itself in a common brain activity. Based on existing data we hypothesized that such commonality might be reflected in mutual activations of a particular brain region. The results show that, indeed, all different kinds of perspective tasks that, to our knowledge, have been used in brain imaging activate the left IPL and precuneus; although the evidence for the latter remains less solid.

(b) Our second achievement was to turn the “overarching view” of a region's broader function, which Cabeza et al. used to summarize existing results, into a predictive instrument. We proceeded in the following way. In a meta-analysis we established that activations of three kinds of perspective tasks show triple overlap in the left IPL and precuneus. This result establishes that the left IPL and precuneus, qualify as areas with the overarching function of tracking perspective. To test the general validity that these regions are responsible for tracking perspective we looked for further perspective tasks. We found several single studies, too few for a meta-analysis. We then needed to check whether the reported activations overlap with the meta-analytic areas, ideally within the area of triple overlap. However, results from single studies do not show the stability of meta-analyses and total overlap with all three tasks from the meta-analysis would be unreasonably conservative. So we settled for the following criterion: The results satisfy the expectations from the overarching view if the activations are found in the target areas (the left IPL and precuneus) and overlap with at least one of the meta-analytic activations in those areas.

With this procedure we were able to show that existing data conform to the hypothesis that the left IPL and precuneus qualify as areas with the overarching function of tracking perspective. We then used the same technique for prediction of identity statements, which qualify as perspective tasks on the basis of a technical account, activate within the overarching regions of the left IPL and precuneus. This prediction was confirmed and with it the hypothesis that these areas track perspective.

The concept of an overarching function helps with the problem of low power of individual studies. For instance, the lack of overlap of activations in our two identity studies can be explained by two factors accounted for in the overarching view. Due to their low power, activations happen to be detected at different points within the overarching region. Another reason for the discrepancy is that the belief revision induced in our first study drew the center of activation more toward the region where false beliefs are processed than in the second study where no belief revision occurred.

### Relation to competing theories

The main competitor for our claim that the left IPL has the overarching function of tracking perspective is the BUA (bottom-up attention) model for the ventral part of the parietal cortex (VPC = IPL) put forward by Cabeza et al. (2012). As an extension of Corbetta and Shulman (2002) dual attention model BUA sees the VPC (IPL) bilaterally responsible for detecting salient and behaviorally relevant stimuli in the environment, especially when they were previously unattended (exogenous, or stimulus-driven attention). Cabeza et al. (2012) extended this model from attention capture by environmental stimuli to capture by internal (memory-based) information. Three interesting aspects arise about the relationship between BUA and perspective tracking: similarities, reducibility, and differences.

#### Similarities

Perspective tasks can be seen as a special kind of internal attention capture. In our thinking and conversations we usually stick to a single perspective because mixing different perspectives is a source of confusion^11^. Therefore, (external or internal reasoning) cues that indicate the need for a change in perspective are exogenous stimuli, and should activate the IPL according to BUA. Attention capture by cues for potential perspective differences is, however, special as it does not require reorienting attention to information about a new topic but reorienting to a new way of informing about (view, mode of presentation, perspective of) the same topic. On these grounds we may consider two possible views of how activation of the left IPL by perspective tasks relates to BUA.

#### Differences

Perspective tracking differs strikingly from BUA in terms of lateralization. Perspective tracking evidently has regional specificity only for the left IPL, while BUA is claimed to operate bilaterally. Cabeza et al. (2012) noticed a prevalence of the left IPL (VPC) activation reports for some tasks in their review and give two possible reasons for it. Left activation reports prevail when predominantly verbal stimulus material is used. However, this explanation does not quite fit the finding that false belief vignettes, which are purely verbal, activate bilaterally (Schurz et al., 2014) while visual perspective tasks, which use a much stronger visual presentation mode, activate exclusively on the left side (Schurz et al., 2013).

Cabeza et al. also suggested that authors often focus on one hemisphere for historical reasons linked to work on patients with lesions, e.g., neglect being observed with right hemisphere parietal lesions. This explanation does not apply to the evidence from perspective tasks we have reviewed, which stems exclusively from fMRI studies without any historical bias. Although few studies test for hemispheric asymmetry the sheer number of studies that report activation only in left and not in the right IPL is remarkable. Of the 14 visual perspective tasks included in the meta-analysis by Schurz et al. (2013) all of them reported activity in left, only Wraga et al. (2010) found bilateral IPL activation. Similarly in our meta-analysis of 16 remember-know studies all of them report left and only Eldridge et al. (2000) reported bilateral IPL activation. This is clear evidence of stronger activation in the left, as only two out of thirty studies (combined vPT + EM) showed bilateral activation and no other study showed activation in the right hemisphere (binomial test *z* = −4.56, *p* < 10^−6^).

Moreover, the two false sign studies (Perner et al., 2006; Aichhorn et al., 2009) only showed effects in the left IPL, and our two studies with identity statements also showed significant reliable activation in the left IPL^12^. The noticeable exception to this left asymmetry are false belief vignettes, which activate the TPJ (including the IPL) on the right as much as on the left (Schurz et al., 2014; see our Figure 1). One reason for this may be that the false belief task engages theory of mind, which activates areas in temporal lobe immediately adjacent and overlapping with the left and right IPL. In contrast, the other perspective tasks show no activations in adjacent areas, only in rather distant areas. All of them tend to activate the precuneus in an overlapping fashion (see Figure 2). Episodic remembering activates bilateral para-hippocampal gyrus areas [e.g., Daselaar et al., 2006; our episodic memory meta-analysis (see Figure 1)], whereas visual perspective tasks activate, the precuneus, left IPL, precentral, and middle frontal region.

#### Reducing perspective tracking to BUA

As outlined above perspective tasks can be seen as a special case of exogenous attention capture, because endogenous thinking usually maintains to the same perspective. One obvious exception to this occurs when perspective itself becomes the topic of thinking. For instance, in visual perspective tasks the instructions are to judge how another viewer sees the display. So taking the other person's perspective is endogenous to the set task and should, according to BUA, activate dorsal parts of the parietal cortex and not the IPL. Another problem case for BUA is a fact persistently ignored in the discussion of why theory of mind tasks activate the TPJ (or IPL) as a consequence of attention reorienting in false belief tasks (Decety and Lamm, 2007; Corbetta et al., 2008; Mitchell, 2008; Cabeza et al., 2012). It is never made clear why the act of reorienting plausibly required in the false belief vignettes (shifting attention from where an object actually is to where an agent mistakenly thinks it is) is not also required in the photo control vignettes (shifting from where the object actually is to where it is in a photo), a contrast introduced by Saxe and Kanwisher (2003) and since used in many studies with exceedingly strong meta-analytic effects (Schurz et al., 2014).

These two problem cases for BUA can be explained by perspective tracking. Visual perspective tasks require perspective tracking hence activate the left IPL. False belief tasks do so too and reliably activate the left IPL, while the photo control tasks do not. A photo taken of the ice cream van in an earlier location does not give a different perspective on where the van is now (unlike a false belief or a flipped direction sign which does give a different view of where the van is now). In sum, although perspective tracking shows a close affinity to bottom-up attention processes it is unlikely that the activation in the left IPL perspective tasks can be completely explained by BUA.

#### Reducing left lateralized BUA to perspective tracking

A different view on perspective tracking and BUA is to claim that only perspective tracking is the overarching function of (at least) the left IPL. To defend this view one would need to show that the evidence recited by Cabeza et al. (2012) in favor of BUA can also be used as evidence for perspective tracking, i.e., that all the tasks that activate the left IPL can be argued to be perspective tasks. Up to now we have considered only tasks that had been independently claimed to be perspective tasks in the developmental literature. Hence, whether a task should or should not activate the left IPL was a predictive enterprise from an existing classification. To retrospectively decide whether a task, which activates the IPL, is a perspective task or not is a much more unconstrained enterprise. We will therefore restrain our analysis to some exemplary illustrations taken from the categories discussed by Cabeza et al.

#### Number processing

Equations can be viewed as identity statements (numerical facts: 4 + 5 is identical to 9) or computational procedures (if you have 4 and add 5 you get 9). So retrieval of numerical facts should activate the left IPL since an identity is likely involved which induces perspective tracking. And, indeed, the IPL is being activated (Dehaene et al., 2003). In contrast, calculation of the result should not activate the IPL or, at least, less so. This also turns out to be the case (Grabner et al., 2009). So, some findings in this area clearly relate to perspective tracking.

#### Episodic retrieval

In contrast to three contenders discussed by Cabeza et al. BUA can explain a characteristic U-function of recognition certainty. The IPL activation is stronger for items judged “definitely old” or “definitely new” than for uncertain answers (data only for the left IPL; Yonelinas et al., 2005; Daselaar et al., 2006). This activation pattern can also result from perspective tracking. Correct recognition can come about for two reasons at least (Jacoby, 1991). One can make a conscious judgment of whether the presented test item has been on the learning list. In some of these cases one may use an episodic approach (Tulving, 1989) and try to re-experience ones' earlier experience of having seen this item during learning. Re-experience requires awareness that one's re-experience of seeing the item is a representation, which gives a perspective, of the past event (Perner et al., 2007). Plausibly if this approach gives a clear answer it will provide high confidence that the item has or has not been experienced. Since awareness of perspective is involved, the confident judgments will activate the left IPL. In other cases no clear judgment may be possible but one can still rely on a feeling of familiarity. Depending on the strength of this feeling one will respond with “old” or “new,” but the subjective confidence will be low. Familiarity judgments do not need awareness of perspective; hence the resultant low confidence answers will not be associated with activations of the left IPL.

## Conclusion

Tracking and monitoring perspectives is a skill whose acquisition has important consequences on children's reasoning and social competence around the age of 4 years. In a meta-analysis of brain imaging in adults we were able to show that this important developmental factor is also reflected in a common cerebral resource: the left IPL and precuneus track perspective. In two empirical studies we were able to extend this finding and confirm that these brain regions are reliably involved in other and novel kinds of perspective tasks, e.g., processing identity statements.

## Author contributions

AA was responsible for Study 3, contributions to the meta-analyses of episodic remembering, and the coordination of all contributions and writing of the manuscript. BW conducted Study 2 in partial fulfillment of his master's degree at the Department of Psychology, University of Salzburg. RW performed the analysis of episodic memory studies. MS provided the general meta-analytic expertise and MA the technical support for collecting and analysing the fMRI data of both studies. JP provided the theoretical framework.

### Conflict of interest statement

The authors declare that the research was conducted in the absence of any commercial or financial relationships that could be construed as a potential conflict of interest.

## Footnotes

^1^With “tracking perspective differences” or, for short, “perspective tracking” we want to merely grasp the existence of this concept required for registering an actual or potential conflict between perspectives. The more common term “perspective taking” suggests the ability to put oneself into another perspective than the perspective one currently has. This would require the tracking of a particular perspective not just the tracking of a potential perspective difference. For, one can be aware of perspectives being involved without being able to switch between them. One can be aware that another person has or may have a different perspective without actually being able to figure out what that perspective is.

^2^With IPL we denote the inferior parietal lobe consisting of the ventral region comprised by BA 40 located in the supramarginal gyrus and BA 39 located in the angular gyrus (Caspers et al., 2006, 2008).

^3^The common name for this task is an unfortunate misnomer because the photo correctly represents the object's earlier location (Perner and Leekam, 2008).

^4^In order to find enough studies to allow for a meta-analysis, Schurz et al. (2013) included level 1 as well as level 2 perspective tasks. Although this is conceptually less than optimal, a follow-up review by the authors showed that the main areas for vPT (e.g., the left IPL and precuneus) were equally often reported in Level 1 and in Level 2 tasks (see Table 3 on p.7 in Schurz et al., 2013). Although level 1 tasks are easy for children because they can be solved without understanding different views of the same target (simply by judging whether the object is within or outside the other person's field of vision), the same activations by level 1 and level 2 tasks in the meta-analysis suggest that level 1 tasks trigger level 2 perspective thoughts in adults. The level 1 question of what the other *sees* tends also to activate concerns about *how* the other sees the object, a level 2 concern.

^5^Anderson and Hastie (1974) showed in a reaction time experiment that people who have learned seemingly about two people and then learn that they are the same person keep the representation (files) for each person separate at first and later tend to merge them into a single file.

^6^Ideally the control sentence should be improved in two ways. One improvement would be to use the same names as in the identity statements: “The tour guide also has a driver,” but that would clash with the first sentence. However, this difference in name is expected to be controlled for by the use of many different sentences using different names for the identity and the control. However, it leaves a systematic difference; the two names mentioned in S1 are both mentioned again in S2 in +IDENT but only one of them in −IDENT. We therefore verified whether repetition of names might activate the left IPL and precuneus in our study. Almor et al. (2007) contrasted a condition where the name introduced in the first sentence was repeated in the second sentence with a condition where a pronoun was used in the second sentence instead. This contrast did not show any activation in the IPL. There was activation in the precuneus, but in quite a different part than the activations in the present study. Another improvement would be to use “is” instead of “has,” e.g., “the tour guide is a driver,” but this creates the danger that participants might gloss this statement as an identity statement and annihilate any activation difference between identity and control condition.

^7^As one of our reviewers rightly pointed out the contradiction in the control task (+REVISION) is a direct incompatibility between S1 and S2, while the contradiction in the identity task only occurs due to natural pragmatic assumptions about S1 of there being two separate individuals. On this basis one would expect stronger activations for belief revision in the control than in the identity task. This safeguards against false positives, i.e., that we would not detect the effects of belief revision in the control task when it is present in the identity task.

^8^This is a novel finding with interesting implications. Attribution of false beliefs to oneself could be a reason why invalid cue trials on the Posner task activate the belief attribution region in the TPJ (Mitchell, 2008).

^9^Although not impossible; one could gloss it as “The lawyer is called Mr Müller.” Also, the use of a proper name in the identity condition raises the potential danger that the proper name is responsible for the left IPL and precuneus activation and not the identity statement itself. Fortunately, existing data from clinical and imaging studies speak against this possibility (for review see Semenza, 2011). Processing of proper names compared to common names was linked to activation in bilateral temporal poles, and, somewhat less consistently, to anterior parts of the superior temporal sulcus, ventral mPFC, and the anterior cingulate. In contrast, the left IPL and precuneus were not associated with processing of proper names.

^10^This finding poses two questions for us. The first one is problematic for our account: Why does this contrast not activate in the left IPL? We can offer the following two post-hoc explanations. Intuitively, in the no-context condition (see Table 5) “The neurologist is Dr. Phillips,” can plausibly be glossed as “The neurologist is called Dr. Phillips,” hence no identity is expressed, and consequently no left IPL activation. This gloss is intuitively less likely when a context is provided: S1 “The doctor saves the lawyer after the accident,” followed by S2 “The lawyer is Mr. Moser,” is less likely to be glossed in a similar way as indicated by the fact that a glossed version of sentence 2 “The lawyer is called Mr. Moser,” would provide an unexpected and less informative content than the un-glossed original version. Our second explanation pertains to the fact that the comprehension questions in the context conditions varied. They could be, e.g., “Who saved the lawyer?,” “Who did the doctor save?,” or “Who is Mr. Moser?” which can only be answered if sentences S1 and S2 have been integrated within a model. In contrast, sentences S2 without context, e.g., “The neurologist is Dr. Phillip,” the questions were always about the person mentioned in S2, “Who is the neurologist?” or “Who is Dr. Phillip?” This question could be answered on the basis of the sentence's surface form without interpreting it within a mental model, i.e., without thinking of different individuals and identity—hence no left IPL activation. The second question raised by this finding is: Why does this contrast activate five areas which are not activated by the sentences in the context conditions? This is an interesting question but not directly problematic for our account. One feature that distinguishes IDENTo > BL from IDENTc > PREDc contrast is that the former contrast is confounded with a contrast of person vs. no person, which could account for at least some of these activations.

^11^A good example are conceptual pacts (Brennan and Clark, 1996) which help ensure that a particular object of conversation is referred to under the same label, since a change of label also entails a change of perspective (Clark, 1997).

^12^However right IPL activation for the identity only condition suggests that the lateralization is one of degree and not one without any involvement of the right hemisphere.

## Abbreviations

+IDENT: identity condition
−IDENT: control of identity condition
+REVISION: belief revision condition
−REVISION: control of belief revision condition
IDENTc: identity-with-context
PREDc: predication-with-context
C: context-only
IDENTo: identity only
BL: baseline condition
FB: false belief
vPT: visual perspective taking
EM: episodic memory.
