# Supplementary material for: Left inferior-parietal lobe activity in perspective tasks: identity statements
Source: Front Hum Neurosci. 2015 Jun 30;9:360. doi: 10.3389/fnhum.2015.00360 (PMC4485079; doi:10.3389/fnhum.2015.00360)
Supplement: Supplementary file 1 [file Table1.DOCX]

**Table S1.**

Recollection > Know/High confidence familiarity- Main characteristics of the tasks in episodic memory meta-analysis.

| **Author** | **Img.** | **Experimental Task** |  | **Control Task** |
| --- | --- | --- | --- | --- |
| Daselaar  2006 | fMRI  n=24 | Participant had to make old/new judgment on the presented words. |  | Participants were prompted to report their confidence for their answer. |
|  |  |  |  |  |
| Denkova  2006 | fMRI  n=12 | Participant were asked to make remember judgment about names, if they can recall any specific personal event associated with the name |  | Participant had to make know judgment, if they can know the identity, but cannot recall any specific event associated with the famous person. |
|  |  |  |  |  |
| Dӧrfel  2009 | fMRI  n=30 | Participant were prompted to make remember judgment for the items within 2 sec. |  | Participant were prompted to make know judgment for the items within 2 sec. |
|  |  |  |  |  |
| Duarte  2007 | fMRI  n=44 | Participants were presented with the items in the study period and were asked to make remember judgment in the test period. |  | Participants were asked to make familiar judgment. |
|  |  |  |  |  |
| Eldridge  2000 | fMRI  n=11 | Participant had to make remember judgment on the studied items. |  | Participant had to make familiar judgment on the studied items. |
|  |  |  |  |  |
| Henson  1999 | fMRI  n=9 | Indicate whether they consciously recollected seeing the word in the previous study. |  | Indicate know judgment that the word was seen in the previous study, but could not recollect any contextual information. |
|  |  |  |  |  |
| Johnson  2007 | fMRI  n=16 | Participants were asked to make remember judgment based on any detail about the word’s study presentation could be recollected. |  | Make know judgment based on the word appeared in the study, but no details could be recollected. |
|  |  |  |  |  |
| Kafka  2012 | fMRI  n=15 | Participants were instructed to report the item as recollected, when the stimulus triggered the recovery of specific details from the study episode. |  | Participant were asked to make familiar judgment, but not engage in effortful recollection but are instructed to report inadvertent recollection. |
|  |  |  |  |  |
| Milton  2011 | fMRI  n=10 | Participant had to distinguish between high and low level of details to make the remember judgment. |  | Participant had to distinguish between high and low familiarity to make know judgment. |
|  |  |  |  |  |
| Montaldi  2006 | fMRI  n=13 | Participants were asked to make remember judgment picture of scenes. |  | Participants were asked to make levels of familiarity judgment. |
|  |  |  |  |  |
| Sharot  2004 | fMRI  n=13 | Participants made remember judgment on the emotional and neutral images presented outside the scanner |  | Participants were asked to make know judgment on the images presented outside the scanner. |
|  |  |  |  |  |
| Smith  2011 | fMRI  n=16 | Participant made old/new recognition judgment based on rating scale, if the word was recollected. |  | Participants were told they should use the know response if they thought the word was familiar but could not recollect any details. |
|  |  |  |  |  |
| Taylor  2013 | fMRI  n=22 | Participants were asked to indicate old/new judgment for the word appeared previously in the study trials. |  | Participants were asked to make know judgment, if they knew the word appeared in the study trials but cannot recollect any details. |
|  |  |  |  |  |
| Vilberg  2007 | fMRI  n=14 | Participants were asked to make remember response if the item paired with the test picture could be recollected. Participants were also required to make remember response when the test item elicited recollection of details of the study episode but not the associated picture. |  | Know response was required when the test picture was judged to be old, and no details could be recollected about the encoding episode. |
|  |  |  |  |  |
| Vilberg  2009 | fMRI  n=18 | Participants were asked to make remember response when some specific aspect of what happened at the time of initial study was recollected. |  | Participants were instructed to make know judgment when nothing could be recollected about the pictures, but confident about the test picture had been studied. |
|  |  |  |  |  |
| Yonelinas  2005 | fMRI  n=16 | Participants were asked to make remember response, if they could recollect anything specific about experiencing the item. |  | Participants were asked to make familiar response if they thought the item had been studied but not entirely sure. |
