# Supplementary material for: Left inferior-parietal lobe activity in perspective tasks: identity statements
Source: Front Hum Neurosci. 2015 Jun 30;9:360. doi: 10.3389/fnhum.2015.00360 (PMC4485079; doi:10.3389/fnhum.2015.00360)
Supplement: Supplementary file 2 [file Table2.DOCX]

**Table S2.**

Study 2 stimulus sentences

|  | Context Sentence | Condition Sentence (+id) | Condition Sentence (-id) | Condition Sentence (+br) | Condition Sentence (-br) |
| --- | --- | --- | --- | --- | --- |
| 1 | Im Petersbrunnhof verdient der Koch so viel wie der Kellner. | Der Koch ist auch der Kellner. | Der Koch hat auch einen Lehrling. | Heute verdient der Kellner mehr als der Koch. | Außerdem arbeitet der Koch so lang wie der Kellner. |
| 2 | Im Hotel Stein arbeitet der Barkeeper so lang wie der Somelier. | Der Barkeeper ist auch der Somelier. | Der Barkeeper hat auch einen Lehrling. | Heute arbeitet der Somelier länger als der Barkeeper. | Außerdem verdient der Barkeeper so viel wie der Somelier. |
| 3 | Bei Red Bull Salzburg hat der Trainer so viel zu sagen wie der Präsident. | Der Trainer ist auch der Präsident. | Der Trainer hat auch einen Assistenten. | Heute hat der Präsident mehr zu sagen als der Trainer. | Außerdem leistet der Trainer so viel wie der Präsident. |
| 4 | Bei Rapid Wien leistet der Kapitän so viel wie der Finanzier. | Der Kapitän ist auch der Finanzier. | Der Kapitän hat auch einen Assistenten. | Heute leistet der Finanzier mehr als der Kapitän. | Außerdem hat der Kapitän so viel zu sagen wie der Finanzier. |
| 5 | An der FH hat der Dekan so viel zu sagen wie der Professor. | Der Dekan ist auch der Professor. | Der Dekan hat auch einen Stellvertreter. | Heute hat der Professor mehr zu sagen als der Dekan. | Außerdem leistet der Dekan so viel wie der Professor. |
| 6 | An der Uni leistet der Doktorand so viel wie der Dozent. | Der Doktorand ist auch der Dozent. | Der Doktorand hat auch einen Diplomanden. | Heute leistet der Dozent mehr als der Doktorand. | Außerdem hat der Doktorand so viel zu sagen wie der Dozent. |
| 7 | In der Hypo hat der Börsenexperte so viel zu sagen wie der Manager. | Der Börsenexperte ist auch der Manager. | Der Börsenexperte hat auch einen Praktikanten. | Diesmal hat der Manager mehr zu sagen als der Börsenexperte. | Außerdem verdient der Börsenexperte so viel wie der Manager. |
| 8 | In der Raiffeisen verdient der Kreditexperte so viel wie der Direktor. | Der Kreditexperte ist auch der Direktor. | Der Kreditexperte hat auch einen Praktikanten. | Diesmal verdient der Direktor mehr als der Kreditexperte. | Außerdem hat der Kreditexperte so viel zu sagen wie der Direktor. |
| 9 | Am Landestheater ist der Darsteller so engagiert wie der Regisseur. | Der Darsteller ist auch der Regisseur. | Der Darsteller hat auch einen Statisten. | Jetzt ist der Regisseur engagierter als der Darsteller. | Außerdem ist der Darsteller so bekannt wie der Regisseur. |
| 10 | An der Staatsoper ist der Tenor so bekannt wie der Dirigent. | Der Tenor ist auch der Dirigent. | Der Tenor hat auch einen Statisten. | Jetzt ist der Dirigent bekannter als der Tenor. | Außerdem ist der Tenor so engagiert wie der Dirigent. |
| 11 | Auf der Busreise spricht der Reiseleiter so viel mit den Gästen wie der Fahrer. | Der Reiseleiter ist auch der Fahrer. | Der Reiseleiter hat auch einen Animateur angestellt. | Heute spricht der Fahrer mehr mit den Gästen als der Reiseleiter. | Außerdem arbeitet der Reiseleiter so lang wie der Fahrer. |
| 12 | Auf der Schiffsreise arbeitet der Organisator so lang wie der Kapitän. | Der Organisator ist auch der Kapitän. | Der Organisator hat auch einen Alleinunterhalter angestellt. | Heute arbeitet der Kapitän länger als der Organisator. | Außerdem spricht der Organisator so viel mit den Gästen wie der Kapitän. |
| 13 | Auf dem Oktoberfest spricht der Ehrengast so lang wie der Bürgermeister. | Der Ehrengast ist auch der Bürgermeister. | Der Ehrengast hat auch einen PR-Berater. | Jetzt spricht der Bürgermeister länger als der Ehrengast. | Außerdem ist der Ehrengast so berühmt wie der Bürgermeister. |
| 14 | Auf dem Ruperti-Kirtag ist der Veranstalter so berühmt wie der Ehrengast. | Der Veranstalter ist auch der Ehrengast. | Der Veranstalter hat auch einen PR-Berater. | Jetzt ist der Ehrengast berühmter als der Veranstalter. | Außerdem spricht der Veranstalter so lang wie der Ehrengast. |
| 15 | Für die WM trainiert der Radfahrer so lang wie der Schwimmer. | Der Radfahrer ist auch der Schwimmer. | Der Radfahrer hat auch einen Trainer. | Heute trainiert der Schwimmer länger als der Radfahrer. | Außerdem ist der Radfahrer so motiviert wie der Schwimmer. |
| 16 | Für Olympia ist der Schütze so motiviert wie der Läufer | Der Schütze ist auch der Läufer. | Der Schütze hat auch einen Trainer. | Heute ist der Läufer motivierter als der Schütze. | Außerdem trainiert der Schütze so lang wie der Läufer. |
| 17 | Am Gymnasium hat der Rektor so viel zu sagen wie der Elternsprecher. | Der Rektor ist auch der Elternsprecher. | Der Rektor hat auch einen Vizerektor. | Dieses Jahr hat der Elternsprecher mehr zu sagen als der Rektor. | Außerdem ist der Rektor so beliebt wie der Elternsprecher. |
| 18 | An der Volksschule ist der Mathelehrer so beliebt wie der Sportlehrer. | Der Mathelehrer ist auch der Sportlehrer. | Der Mathelehrer hat auch einen Referendar. | Dieses Jahr ist der Sportlehrer beliebter als der Mathelehrer. | Außerdem hat der Mathelehrer so viel zu sagen wie der Sportlehrer. |
