# Supplementary material for: Left inferior-parietal lobe activity in perspective tasks: identity statements
Source: Front Hum Neurosci. 2015 Jun 30;9:360. doi: 10.3389/fnhum.2015.00360 (PMC4485079; doi:10.3389/fnhum.2015.00360)
Supplement: Supplementary file 3 [file Table3.DOCX]

**Table S3.**

Study 3 stimulus sentences

|  | Context Sentence |  | Condition Sentence |  | Questions |
| --- | --- | --- | --- | --- | --- |
|  |  |  | PREDc List 1 | IDENTc List 2 |  |
| 1 | Der Töpfer geht mit dem Künstler ein Bier trinken. | Der Künstler | ist gepflegt | ist Herr Lechner |  |
| 2 | Der Passauer zeltet im Wald des Jägers. | Der Passauer | ist muskulös | ist Herr Arndt |  |
| 3 | Die Hausfrau feiert den Geburtstag mit Ihrer Gehilfin. | Die Hausfrau | ist mollig | ist Lea | Wer feiert den Geburtstag mit Ihrer Gehilfin? |
| 4 | Der Flachgauer macht der Tänzerin das Make-up. | Der Flachgauer | ist dick | ist Herr Bittner |  |
| 5 | Der Bremer kaufte ein Auto beim Fachmann. | Der Fachmann | ist alt | ist Herr Schwarz |  |
| 6 | Der Berliner und der Hamburger arbeiten im Supermarkt. | Der Hamburger | ist groß | ist Herr Doppler |  |
| 7 | Der Kölner und der Aachener essen zu Abend. | Der Aachener | ist brünett | ist Benjamin |  |
| 8 | Der Hausmeister und der Arbeiter kaufen sich einen Kebap. | Der Arbeiter | ist tätowiert | ist Uwe |  |
| 9 | Der Koblenzer joggt so oft wie der Trainer. | Der Trainer | ist sportlich | ist Herr Hager | Der Koblenzer joggt so oft wie wer? |
| 10 | Der Klempner trifft den Kasseler auf der Baustelle. | Der Klempner | ist flink | ist Dr. Kuhn | Wer ist flink? |
| 11 | Der Detektiv verfolgt den Schriftsteller. | Der Schriftsteller | ist bucklig | ist Herr Ebner |  |
| 12 | Der Erdinger singt im Chor des Landsbergers. | Der Landsberger | ist rundlich | ist Herr Reiter | Der Landsberger ist was? |
| 13 | Der Würzburger bekommt eine Behandlung vom Zahnarzt. | Der Würzburger | ist verschnupft | ist Herr Mayer |  |
| 14 | Der Notar und der Bürokrat entgehen knapp einem Verkehrsunfall. | Der Notar | ist mittleren Alters | ist Herr Adler | Der Notar ist welchen Alters? |
| 15 | Der Prager und der Tessiner gehen in die Kirche. | Der Tessiner | ist gut aussehend | ist Dr. Felber | Wer geht mit dem Prager in die Kirche? |
| 16 | Der Arzt rettet den Anwalt nach einem Unfall. | Der Anwalt | ist jung | ist Herr Wagner | Wer rettet den Anwalt nach einem Unfall? |
| 17 | Der Wormser und der Konstanzer arbeiten im Labor. | Der Wormser | ist langsam | ist Herr Lessing |  |
| 18 | Der Telefonist und der Vertreter treffen sich auf einer Party. | Der Telefonist | ist gehbehindert | ist Herr Huber | Wen trifft der Vertreter auf der Party? |
| 19 | Der Friseur geht mit dem Optiker Mittagessen. | Der Optiker | ist blass | ist Herr Schmidt |  |
| 20 | Der Salzburger geht Skifahren mit dem Laufener. | Der Salzburger | ist rothaarig | ist Paul |  |
| 21 | Der Juwelier ist ein Freund des Architekten. | Der Juwelier | ist kraushaarig | ist Herr Dietrich |  |
| 22 | Der Halleiner und der Münchner fahren Rad. | Der Münchner | ist sonnengebräunt | ist Herr Aigner | Wer fährt mit dem Münchner Rad? |
| 23 | Der Masseur nimmt Tanzunterricht bei der Welserin. | Der Masseur | ist schmächtig | ist Herr Pichler |  |
| 24 | Der Angestellte und der Sekretär gehen wandern. | Der Angestellte | ist gepierct | ist Dr. Eder |  |
| 25 | Der Schlosser arbeitet mit dem Dachdecker zusammen. | Der Dachdecker | ist stark | ist Peter |  |
| 26 | Der Fotograf ruft den Redakteur an. | Der Fotograf | ist dunkelhäutig | ist Herr Becker |  |
| 27 | Der Kommissar trinkt noch einen Kaffee mit dem Fuschler. | Der Kommissar | ist verletzt | ist Herr Moser |  |

|  | Context Sentence |  | Condition Sentence |  | Questions |
| --- | --- | --- | --- | --- | --- |
|  |  |  | IDENTc List 1 | PREDc List 2 |  |
| 1 | Der Bonner und der Mainzer arbeiten im Hotel Castellani. | Der Bonner | ist Herr Mayer | ist verschnupft |  |
| 2 | Der Florist hilft dem Förster mit den Pflanzen. | Der Förster | ist Leo | ist mollig |  |
| 3 | Der Grazer und der Innsbrucker arbeiten bei der Polizei. | Der Innsbrucker | ist Herr Moser | ist verletzt | Mit wem arbeitet der Grazer bei der Polizei? |
| 4 | Der Assistent fand den Rucksack des Darmstädters. | Der Assistent | ist Herr Arndt | ist muskulös |  |
| 5 | Der Tischler und der Pinzgauer haben frei. | Der Tischler | ist Peter | ist stark |  |
| 6 | Der Techniker arbeitet am Projekt des Informatikers. | Der Informatiker | ist Uwe | ist tätowiert |  |
| 7 | Der Komiker ist mit dem Autor einen Cocktail trinken. | Der Autor | ist Herr Schmidt | ist blass |  |
| 8 | Der Flensburger wird von dem Psychologen betreut. | Der Flensburger | ist Herr Reiter | ist rundlich |  |
| 9 | Der Kellner serviert dem Ulmer das Essen. | Der Kellner | ist Herr Schwarz | ist alt |  |
| 10 | Der Pilot trifft im Fitnessstudio den Beamten wieder. | Der Pilot | ist Herr Huber | ist gehbehindert |  |
| 11 | Der Bayer und der Walser sitzen im Bus. | Der Walser | ist Herr Lechner | ist gepflegt |  |
| 12 | Der Leipziger und der Duisburger sind in einer Besprechung. | Der Leipziger | ist Dr. Felber | ist gut aussehend |  |
| 13 | Der Maler arbeitet an einem alten Bild des Sammlers. | Der Maler | ist Herr Adler | ist mittleren Alters | Wer arbeitet an einem alten Bild des Sammlers? |
| 14 | Der Assistent wartet auf den Zug des Mannheimers. | Der Assistent | ist Herr Bittner | ist dick |  |
| 15 | Der Makler schreibt an den Gmundner. | Der Gmundner | ist Herr Dietrich | ist kraushaarig | Wer schreibt an den Gmundner? |
| 16 | Der Landshuter hilft dem Bayreuther . | Der Landshuter | ist Herr Aigner | ist sonnengebräunt | Wer ist der Landshuter? |
| 17 | Die Stewardess kennt den Leipziger erst seit kurzem. | Der Leipziger | ist Herr Wagner | ist jung |  |
| 18 | Der Fürther arbeitet bei der Feuerwehr mit dem Giessener zusammen. | Der Fürther | ist Herr Hager | ist sportlich | Wer ist Herr Hager? |
| 19 | Der Bregenzer und der Tiroler geben ein Konzert. | Der Tiroler | ist Herr Becker | ist dunkelhäutig |  |
| 20 | Der Ökonom und der Berner reisen ins Ausland. | Der Ökonom | ist Herr Lessing | ist langsam |  |
| 21 | Der Kärntner entwirft das Haus des Pianisten. | Der Pianist | ist Herr Pichler | ist schmächtig |  |
| 22 | Der Winzer wird mit dem Augsburger zu Mittag essen. | Der Augsburger | ist Dr. Kuhn | ist flink |  |
| 23 | Der Student und der Schweizer meditieren im Garten. | Der Schweizer | ist Herr Doppler | ist groß |  |
| 24 | Der Hofheimer hat sich mit dem Bauherrn gestritten. | Der Hofheimer | ist Paul | ist rothaarig |  |
| 25 | Der Chirurg war ein Klassenkamerad des Dortmunders. | Der Dortmunder | ist Benjamin | ist brünett |  |
| 26 | Der Weizer unterhält sich mit dem Kremser. | Der Kremser | ist Dr. Eder | ist gepierct |  |
| 27 | Das Hausmädchen arbeitet für den Direktor. | Das Hausmädchen | ist Frau Ebner | ist bucklig | Wer arbeitet für den Direktor? |

|  | Context only |  |  |  | Question |
| --- | --- | --- | --- | --- | --- |
| 1 | Der Berliner ist ein Nachbar des Erfurters. |  |  |  |  |
| 2 | Der Mediziner lehrt dem Zeller Yoga. |  |  |  | Wem lehrt der Mediziner Yoga? |
| 3 | Der Gitarrist arbeitet mit dem Londoner an einem Musik-Projekt. |  |  |  |  |
| 4 | Der Schärdinger trifft morgen den Ennser. |  |  |  |  |
| 5 | Der Botschafter wartet mit dem Genfer am Flughafen. |  |  |  | Mit wem wartet der Botschafter am Flughafen? |
| 6 | Der Villacher trifft den Klagenfurter am Schalter. |  |  |  |  |
| 7 | Der Sänger und der Schauspieler wohnen im Hotel Stein. |  |  |  | Wer wohnt mit dem Schauspieler im Hotel Stein? |
| 8 | Der Portier hört die Musik des Marburgers. |  |  |  |  |
| 9 | Der Lehrer bringt dem Füssener das Malen bei. |  |  |  | Wer bringt dem Füssener das Malen bei? |
| 10 | Der Augsburger geht auf die Hochzeit des Briefträgers. |  |  |  |  |
| 11 | Der Bauer gibt dem Knecht Anweisungen. |  |  |  |  |
| 12 | Der Kieler spielt in der Band des Bitburgers. |  |  |  | Wer spielt in der Band des Bitburgers? |
| 13 | Der Braunauer redet mit dem Wirt über die Reparaturen. |  |  |  |  |
| 14 | Der Lindauer ist auf Dienstreise mit dem Juristen. |  |  |  | Mit wem ist der Lindauer auf Dienstreise? |
| 15 | Der Philosoph bringt dem Schüler Logik bei. |  |  |  |  |
| 16 | Der Therapeut geht mit dem Essener auf eine Weltreise. |  |  |  |  |
| 17 | Die Aushilfe sieht sich mit dem Tullner einen Film an. |  |  |  |  |
| 18 | Der Oberst besucht den Soldaten. |  |  |  |  |
| 19 | Der Pongauer trifft im Urlaub den Ischler. |  |  |  | Wer trifft im Urlaub den Ischler? |
| 20 | Der Geologe lud den Starnberger zu seiner Ausstellung ein. |  |  |  | Wer lud den Starnberger zu seiner Ausstellung ein? |
| 21 | Der Matrose rettet den Steyrer auf hoher See. |  |  |  | Wen rettet der Matrose auf hoher See? |
| 22 | Der Feldbacher sieht den Grieskirchner in einer TV-Show auftreten. |  |  |  | Wer sieht den Grieskirchner in einer TV-Show auftreten? |
| 23 | Der Liezener arbeitet im Nationalpark für den Tierpfleger. |  |  |  |  |
| 24 | Der Freistädter borgt sich ein Buch vom Apotheker. |  |  |  |  |
| 25 | Der Verkäufer sitzt im Cafe neben dem Polizisten. |  |  |  | Nebem wem sitzt der Verkäufer im Cafe? |
| 26 | Der Mechaniker und der Linzer gehen zum Musikkonzert. |  |  |  |  |
| 27 | Der Lübecker bucht für den Dresdner ein Hotelzimmer. |  |  |  | Für wen bucht der Lübecker ein Hotelzimmer? |

|  | Identity only |  |  |  | Questions |
| --- | --- | --- | --- | --- | --- |
| 1 | Der Wachauer ist Herr Hofer |  |  |  |  |
| 2 | Der Taucher ist Christian |  |  |  |  |
| 3 | Der Nassauer ist Dr. Weiss |  |  |  |  |
| 4 | Der Manager ist Helmut |  |  |  |  |
| 5 | Der Freiburger ist Laurenz |  |  |  |  |
| 6 | Der Bamberger ist Herr Holzner |  |  |  | Wer ist Herr Holzner? |
| 7 | Der Kapitän ist Florian |  |  |  |  |
| 8 | Der Bäcker ist Herr Tikal |  |  |  |  |
| 9 | Der Pastor ist Dr. Egger |  |  |  | Wer ist Dr. Egger? |
| 10 | Der Altdorfer ist Herr Barth |  |  |  | Wer ist Herr Barth? |
| 11 | Der Metzger ist Andreas |  |  |  |  |
| 12 | Der Imker ist Dr. Keller |  |  |  |  |
| 13 | Der Richter ist Herr Schlager |  |  |  |  |
| 14 | Der Seemann ist Anton |  |  |  |  |
| 15 | Der Buchhändler ist Herr Bauer |  |  |  |  |
| 16 | Der Fahrer ist Herr Wallner |  |  |  |  |
| 17 | Der Bochumer ist Herr Hoffmann |  |  |  |  |
| 18 | Der Weimarer ist Herr Gangl |  |  |  | Wer ist Herr Gangl? |
| 19 | Der Koch ist Dr. Schwab |  |  |  |  |
| 20 | Der Neurologe ist Herr Phillips |  |  |  | Wer ist der Neurologe? |
| 21 | Der Lungauer ist Lukas |  |  |  | Wer ist der Lungauer? |
| 22 | Der Biologe ist Herr Korber |  |  |  | Wer ist der Biologe? |
| 23 | Der Moderator ist Martin |  |  |  | Wer ist der Moderator? |
| 24 | Der Wittener ist Thomas |  |  |  |  |
| 25 | Der Major ist Dr. Danzer |  |  |  | Wer ist der Major? |
| 26 | Der Ansbacher ist Herr Kramer |  |  |  |  |
| 27 | Der Schaffner ist Herr Ebner |  |  |  |  |

|  | Baseline |  |  |  | Questions |
| --- | --- | --- | --- | --- | --- |
| 1 | Die Teekanne ist viel zu heiß |  |  |  |  |
| 2 | Die Karte ist zerknittert |  |  |  | Die Karte ist was? |
| 3 | Die Uniformhose ist eng |  |  |  |  |
| 4 | Das Telefon ist modern |  |  |  |  |
| 5 | Die Thermosflasche ist leer |  |  |  |  |
| 6 | Das Lineal ist dreieckig |  |  |  |  |
| 7 | Der Ring ist schön |  |  |  |  |
| 8 | Die Armbanduhr ist kaputt |  |  |  |  |
| 9 | Der Schnellzug ist beleuchtet |  |  |  | Was ist beleuchtet? |
| 10 | Der Schnellzug ist beleuchtet |  |  |  |  |
| 11 | Der Rahmen ist quadratisch |  |  |  | Was ist quadratisch? |
| 12 | Das Weinglas ist zerbrochen |  |  |  |  |
| 13 | Das Autoradio ist laut |  |  |  |  |
| 14 | Der Geländewagen ist schnell |  |  |  |  |
| 15 | Die Schokolade ist dunkel |  |  |  |  |
| 16 | Die Brille ist altmodisch |  |  |  |  |
| 17 | Die Eingangstür ist versperrt |  |  |  | Was ist versperrt? |
| 18 | Das Haselnussblatt ist grün |  |  |  |  |
| 19 | Der Streuselkuchen ist verziert |  |  |  |  |
| 20 | Das Foto ist eingerahmt |  |  |  |  |
| 21 | Die Schultafel ist sauber |  |  |  |  |
| 22 | Der Stöckelschuh ist schmutzig |  |  |  |  |
| 23 | Das Spielzeugfahrrad ist neu |  |  |  |  |
| 24 | Die Christbaumkugel ist rund |  |  |  |  |
| 25 | Die Schachtel ist achteckig |  |  |  |  |
| 26 | Das Kopierpapier ist dünn |  |  |  |  |
| 27 | Die Geldtasche ist dunkelblau |  |  |  | Was ist die Geldtasche? |
